# Gene expression variations in high-altitude adaptation: a case study of the Asiatic toad (*Bufo gargarizans*)

**DOI:** 10.1186/s12863-017-0529-z

**Published:** 2017-07-03

**Authors:** Weizhao Yang, Yin Qi, Bin Lu, Liang Qiao, Yayong Wu, Jinzhong Fu

**Affiliations:** 1 Chengdu Institute of Biology, Chinese Academy of Sciences, Chengdu, 610041 China; 2 Department of Integrative Biology, University of Guelph, Guelph, ON N1G 2W1 Canada

**Keywords:** High-altitude, Toads, Gene expression, Down-regulation, Nutrient metabolism, Cardiac functions

## Abstract

**Background:**

Genome-wide investigation of molecular mechanisms for high-altitude adaptation has attracted great attention in the last few years. In order to understand the contribution of gene expression level variations to high-altitude adaptation in Asiatic toads (*Bufo gargarizans*), we implemented a reciprocal transplant experiment between low- and high-altitude sites and sequenced 12 transcriptomes from brain, heart, and liver tissues.

**Results:**

A large number of genes with expression differences (DEGs) between high- and low-altitude individuals (193 fixed and 844 plastic) were identified, and the majority of them were tissue specific. Heart displayed the largest number of DEGs, both plastic and fixed. Fixed DEGs were particularly concentrated in functions associated with muscle contraction, and the majority of them were down-regulated in high-altitude individuals. Plastic DEGs were highly concentrated in several energy metabolism related functional categories, and the majority of them were also down-regulated at high-altitude environments. In liver samples, genes associated with nutrient metabolism experienced a broad-scale expression down-regulation in high-altitude toads.

**Conclusions:**

These broadly suppressed expression patterns at high altitudes are in strong contrast to those of endothermic homeotherms, suggesting poikilothermic vertebrates may have adopted different strategies at high altitudes. Our results strongly support that both genotypic specialization and phenotypic plasticity play crucial role in adaptation to high altitude for Asiatic toads. Poikilothermic vertebrates are among the most hypoxia-tolerant animals known, and many molecular mechanisms remain elusive. We hope that our results will provide useful directions for future research.

**Electronic supplementary material:**

The online version of this article (doi:10.1186/s12863-017-0529-z) contains supplementary material, which is available to authorized users.

## Background

High-altitude environments provide an excellent study system for understanding adaptation, and numerous case studies have provided great insights into the mechanisms of evolution [1–4]. At high-altitudes, organisms experience multiple stressors, such as low oxygen, low temperature, and high UV radiation. Simultaneous adaptive responses to these stressors require interactions and trade-offs within the genetic networks, which provides opportunities to explore the complex genetic basis of adaptation [4]. Furthermore, an altitudinal gradient allows large ecological variations within short spatial distance, which offers excellent natural experiments for selection-driven local adaptation [1]. With the recent development of genome technology, investigation of genome-wide molecular mechanisms of high-altitude adaptation has attracted great attention in the last few years [5–10]. These studies not only confirmed many early conclusions from physiological studies, but also identified previously unknown mechanisms [11]. Nevertheless, most of these studies were focused on sequence level variations of protein-coding genes, and few have examined the contribution of gene expression level variations to high-altitude adaptation [12].

Gene expression level variations may derive from mutations in regulatory regions of the genome or environment induced plasticity. The importance of regulatory changes (vs. protein changes) in adaptive evolution has been widely recognized [13–15], although many of its issues remain a topic of debate [16]. It is also well-recognized that environment induced phenotypic plasticity plays an important role in adaptation [17]. Some alterations in phenotypes are formed during an individual’s development (developmental plasticity) and are lasting, while other alterations are physiological plasticity during adulthood that can be reversed in relatively short time. As consequences of these processes, gene expression level variations can be derived from mutations and hence fixed, or can be induced by environments and hence plastic.

Both genotypic specialization and phenotypic plasticity are expected to contribute to high-altitude adaptation [18, 19], and several previous studies examined the gene expression variation at the genomic level. Using common garden experiments, Cheviron et al. [12, 19] and Scott et al. [20] examined genome-wide gene expression patterns of deer mice from various elevations. Most prominently, deer mouse individuals from high altitudes showed a large-scale up-regulation of genes associated with oxygen utilization and metabolic fuel use. Most of these changes occurred over both physiological and developmental timescales. In a similar common garden experiment, Cheviron et al. [21] (2008) also compared gene expression profiles of rufous-collared sparrows from different altitudes and concluded that substantial plasticity may have contributed to high-altitude adaptation. These studies clearly demonstrated that adaptive evolution can proceeds through transcriptional variation and much work is needed in this regard.

We recently initiated a project examining the genetic basis of high-altitude adaptation in Tibetan amphibians. A majority of genetic studies on high-altitude adaptation examined endothermic homeotherms (i.e. mammals and birds). Poikilotherms, such as amphibians, have very different physiology from homeotherms (e.g. low metabolic rates, low and variable body temperatures) and their physiological responses to high-altitude environments are much more variable [22]. Therefore, we expect that poikilotherms have evolved different adaptive genetic mechanisms from homeotherms. The Asiatic toad (*Bufo gargarizans*) is one of the few amphibians that have successfully colonized the Tibetan Plateau, and its distribution range extends an extremely large altitudinal gradient from zero to 4300 m [23]. Its populations from different altitudes display significant phenotypic differences. For example, populations at 2100 m have a slower growth rate and a delayed sexual maturity than populations at 760 m [24]. Our previous study examined protein-coding gene variations and their potential contribution to high-altitude adaptation, and found that variations in genes associated with nutrient metabolism appear to be important while genes related to hypoxia tolerance may not [10]. This marked a significant difference from previous studies on homeothermic vertebrates [5–7].

To gain an understanding of how the gene expression level variations may have contributed to high-altitude adaptation in the Asiatic toad (*Bufo gargarizans*), we implemented a reciprocal transplant experiment between low- and high-altitude sites. The gene expression profiles of four treatment groups were obtained with transcriptome sequencing, and we identified differentially expressed genes (DEGs) between high- and low-altitudes sites with both fixed and plastic variations. We further explored the contribution of these DEGs to high-altitude adaptation and compared to those of homeotherms.

## Methods

### Reciprocal transplant experiment

**Fig. 1:**
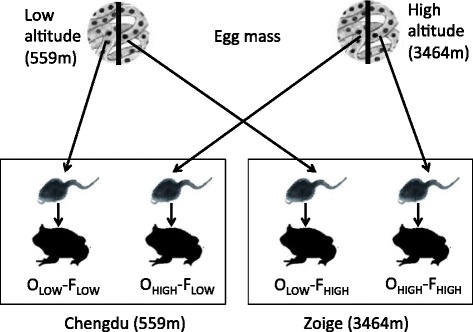
Design of the reciprocal transplant experiment

We implemented a reciprocal transplant and common garden experiment between a low-altitude site (Chengdu; 104.01°E, 30.91°N, 559 m) and a high-altitude site (Zoige; 102.48°E, 33.72°N, 3464 m) with four treatment groups. An egg mass of the Asiatic toad originated from Chengdu (O_LOW_) was collected in February 2014 and randomly separated into two halves. One half was fostered at Chengdu (O_LOW_-F_LOW_) and the other half was fostered at Zoige (O_LOW_-F_HIGH_). Similarly, an egg mass originated from Zoige was collected in April 2014 (O_HIGH_), and randomly separated into two halves. One half was fostered at Zoige (O_HIGH_-F_HIGH_) and the other half was fostered at Chengdu (O_HIGH_-F_LOW_). A diagram depicting the design is presented in Fig. 1.

All tadpoles were exposed to natural local conditions of the foster sites, including photoperiod, ambient temperature, natural sunlight, and air. Extra plant materials (boiled lettuce) were added as food. Other than seasonal differences, fostering conditions were kept as consistent as possible between the treatment groups. The O_LOW_-F_LOW_ group completed metamorphosis from April 27th to May 3rd, and the O_LOW_-F_HIGH_ group completed metamorphosis from July 16 to August 5. The O_HIGH_-F_LOW_ group completed metamorphosis from June 3rd to 7th and the O_HIGH_-F_HIGH_ group completed metamorphosis from August 10 to September 13. One month after metamorphosis, three individuals with similar snout-vent length (1.2–1.4 cm) and body mass (0.2–0.3 g) were selected from each treatment group. All individuals were euthanized by submerging in MS-222 solution (1 g/L). Immediately following euthanasia and dissection, three tissues (brain, liver, and heart) were collected and preserved in Sample Protector (Takara Bio; Shiga, Japan) for gene expression examination.

### Transcriptome sequencing protocols

Gene expression levels in three tissue types (brain, heart, and liver) were measured by transcript abundance using RNA-seq (Additional file 1). RNA was extracted from each tissue sample according to the TRIzol protocols (Invitrogen*;* Carlsbad, California). Since the amount of RNA from a single tissue sample was not enough for Illumina sequencing, we pooled RNAs from the same type of tissue from all three individuals of each treatment group with approximately equal quantity. This procedure allowed us to obtain an average estimate among the individuals but without a standard deviation. Single-end sequencing with read length of 50 base pair was carried out on an Illumina HiSeq2000 platform. Library construction and sequencing were performed by Novogene (Beijing, China).

### Identification for differentially expressed genes

Raw reads were first cleaned by removing the adapter sequences and low-quality base calls using a Novogene pipeline. The short reads were mapped against two assembled transcriptomes from our previous work [10] using Bowtie v1.1.2 [25]. The sequences obtained from eggs originated from Chengdu were mapped to a transcriptome assembly generated from individuals of Chengdu, and sequences obtained from eggs originated from Zoige were mapped to a transcriptome assembly generated from individuals of Zoige. Only high quality orthologous coding sequences were used and reads mapped to multiple genes were discarded. The number of reads matched to each gene was counted by HTSeq-count tool with the “union” resolution mode [26]. The TMM normalization method implemented in edgeR package [27] was used to align expression values to a common scale. Differentially expressed genes (DEGs) were estimated through generalized linear models with paired samples, which suited our experimental design. We adopted a strict criterion to identify DEGs, with fold-value ≥2 and adjusted *P*-value <0.005 (FDR).

We identified two types of DEGs, fixed and plastic. Fixed DEGs are genes that revealed significantly differential expression between individuals from native low- and high-altitude environments (O_LOW_ vs. O_HIGH_), regardless of their foster sites. On the other hand, genes that show significantly differential expression between individuals from foster low- and high-altitude environments (F_LOW_ vs. F_HIGH_) are regarded as plastic [21].

Spearman’s rank correlation coefficient was used to measure the degree of correlation for each tissue type in expression comparisons. The standardized expression values for each group and each tissue were used to calculate the coefficient between O_LOW_ and O_HIGH_ and between F_LOW_ and F_HIGH_. Low correlation coefficient indicated high divergence in expression for a tissue.

### Functional analyses

Annotations of all putative DEGs were obtained from published transcriptome sequences of *Bufo gargarizans* [10]. Functional over-representation of DEGS was performed using the GOStats package [28] in R with annotation to GO category and KEGG pathway database. The minimum number of genes required for each test of a given category was 20. All tests were corrected by false discovery rate (FDR). A cluster analysis was also conducted to identify the functions with significantly redundant annotation terms using the DAVID v6.8 pipeline [29]. All statistical analyses were carried out using R.

## Results

### Differences in global expression

A total of 12 transcriptomes (4 pools × 3 tissues) were sequenced and the numbers of clean reads for each transcriptome ranged from 9.9 million to more than 15 million (Additional file 1). A total of 4684, 4453, and 4521 annotated genes (transcripts) from brain, liver, and heart tissues respectively were mapped to the established transcriptome assemblies. After the mapping, genes with different levels of expression were identified.

**Fig. 2:**
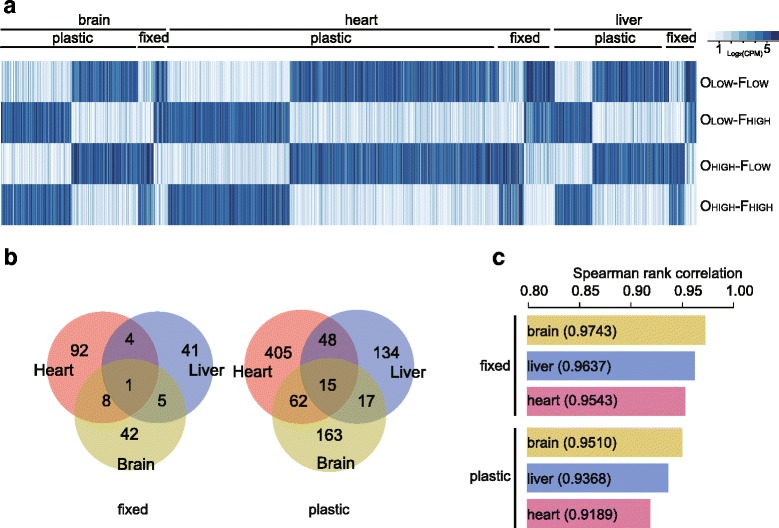
Differentially expressed genes (DGEs) of Asiatic toads at high altitudes. **a**. Heatmaps of gene expression profiles for the four treatment groups. There are more plastic DEGs than fixed ones in any tissue, and the heart has more DEGs than other tissues, both fixed and plastic. **b**. Venn-diagram of fixed and plastic DEGs among three tissues. Most DEGs are tissue-specific. **c**. Spearman rank correlations between O_LOW_ and O_HIGH_ treatment groups (fixed DEGs) and between F_LOW_ and F_HIGH_ treatment groups (plastic DEGs). Correlation coefficients are smaller among plastic DEGs than among fixed DEGs. The heart had the weakest correlation in both comparisons

A total of 193 fixed DEGs and 844 plastic DEGs were detected (Fig. 2a and b; Additional file 2). Expression correlations between O_LOW_ and O_HIGH_ treatment groups and between F_LOW_ and F_HIGH_ treatment groups were estimated for each tissue type (Fig. 2c). Correlation in heart was the weakest among the three tissues, suggesting heart has the most expression differences. Also, correlation coefficients were smaller in the foster-site comparison than in the origin-site comparison, suggesting more variations were caused by environment (plastic) than by mutation (fixed).

### Tissue-specific expression differences

**Fig. 3:**
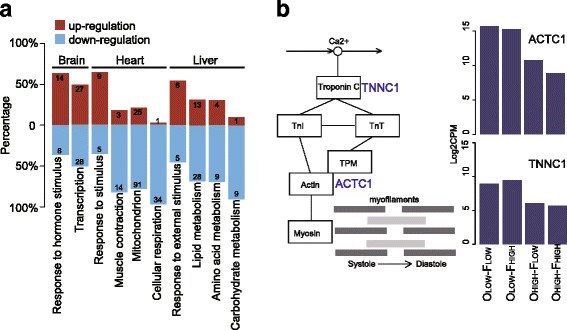
Related functions of differentially expressed genes (DEGs). **a**. Expression patterns of DEGs by main functional clusters and tissue type for O_HIGH_-F_HIGH_ treatment group. DEGs in stimulus response related clusters show a clear up-regulation trend; DGEs related to nutrient metabolism in the liver and DGEs related to muscle contraction and cellular respiration in the heart exhibit a clear broad-scale down-regulation. The numbers in each bar are numbers of genes. **b**. The positions of two core genes, ACTC1 and TNNC1, within the cardiac contraction pathway, and their expression levels in the four treatment groups

A total of 18 fixed and 142 plastic DEGs were differentially expressed in at least two tissue types (Fig. 2b). MED24 was the only gene that showed fixed differences in all three tissues, and all were down-regulated for high-altitude individuals. MED24 is an upstream transcriptional factor for many genes [30]. Over-representation analysis revealed that GO category *response to stimulus* and its several child categories were over-represented by DEGs in all three tissues (Fig. 3a). The DEGs related to stimulus response showed an up-regulating trend in comparison with other major functional clusters (one-tailed Fisher’s exact test, *P* = 2.20E-6), suggesting that environmental stimulus are likely a common stress to all tissues at high altitudes. Nevertheless, The majority of DEGs were differentially expressed in only one tissue (Table 1), indicating each tissue may play a specific role in coping with the challenging environments.

**Table 1:** Tissue-specific gene expression patterns in high-altitude Asiatic toads (*Bufo gargarizans*), compared to low-altitude individuals. Fixed = comparison between O_LOW_ vs O_HIGH_; Plastic = comparison between F_LOW_ vs F_HIGH_

	Fixed		Plastic		Total
	Down-regulated	Up-regulated	Down-regulated	Up-regulated	
Liver	22 (43.1%)	29 (56.9%)	144 (67.3%)	70 (32.7%)	265
Brain	27 (48.2%)	29 (51.8%)	125 (48.6%)	132 (51.4%)	313
Heart	58 (55.2%)	47 (44.8%)	302 (57.0%)	228(43.0%)	635

Heart had the largest number of DEGs among the three tissue types (Fig. 2b, Table 1), both fixed (105) and plastic (530). Fixed DEGs were mainly concentrated in muscle contraction related GO categories (besides the *response to stimulus* category), such as *muscle system process*, *muscle contraction*, and *striated muscle contraction* (Additional file 2). Remarkably, among the 17 fixed DEGs associated with muscle contraction, 14 were down-regulated in high-altitude individuals (Fig. 3a). In particular, two core genes within cardiac muscle contraction pathway, ACTC1 and TNNC1, were down-regulated. These two genes encode core subunits of actin and troponin, which directly feature in the cardiac contraction process (Fig. 3b). Furthermore, the myosin related gene, MYBPC2, was also down-regulated in heart. Plastic DEGs were highly concentrated in a large number of energy metabolism related GO categories, such as *cellular respiration*, *generation of precursor metabolites energy*, *electron transport chain*, and *mitochondrial part* (Additional file 2). Mitochondrion is the organelle where energy metabolism takes place, and 91 (out of 116) plastic DEGs associated with category *mitochondrion* were down-regulated for high-altitude individuals (Fig. 3a). In addition, 34 (out of 35) DEGs related to category *cellular respiration* exhibited down-regulation at high altitudes (Fig. 3a).

In liver, 265 DEGs were detected with 51 being fixed and 214 being plastic (Table 1). The functional spectrum of fixed and plastic DEGs was similar and highly concentrated in nutrient metabolism (Additional file 2). Among all DEGs associated with nutrient metabolism, a majority were down-regulated in the O_HIGH_-F_HIGH_ treatment group, including 28 of 41 genes function in lipid metabolism, 9 of 13 genes function in amino acid metabolism, 9 of 10 genes function in carbohydrate metabolism (Fig. 3a). Similar broad down-regulation was also observed in several regulatory pathways, including *fatty acid degradation*, *glycolysis and gluconeogenesis*, and *insulin signaling*.

In brain, 313 DEGs were detected with 56 being fixed and 257 being plastic (Table 1). The functions of fixed and plastic DEGs were both primarily clustered in transcriptional regulation (Additional file 2). For example, 55 DEGs were related to transcriptional factors, and 28 were down-regulated and 27 were up-regulated for the O_HIGH_-F_HIGH_ group (Fig. 3a). Similarly, 22 plastic DEGs were associated with the GO category *response to hormone stimulus*, of which 8 were down-regulated and 14 were up-regulated for toads at high altitudes (Fig. 3a).

## Discussion

Our reciprocal transplant experiment and gene expression analysis clearly demonstrated that both plastic and fixed gene expression variations have contributed to high-altitude adaptation in Asiatic toads (*Bufo gargarizans*). We identified a large number of genes with plastic and fixed expression differences between high- and low-altitude individuals. Furthermore, the majority of these DEGs showed suppressed expression at high-altitude environments.

Genes associated with nutrient metabolism experienced a broad-scale expression down-regulation in toads living at high-altitude environments. This includes both fixed and plastic DEGs and is most prominently displayed in liver (Fig. 3a). This observation represents perhaps the most striking difference from endothermic homeotherms. Similar transplant studies of collared sparrow [21] and deer mice [12] both exhibited an acceleration of nutrient metabolism at high-altitudes by up-regulating a series of related genes. For example, genes involved in fatty acid oxidation and OXPHOS genes had a clear trend of up-regulation in highland deer mice [12]. Our previous work on protein-coding gene sequences of Asiatic toads also detected positive selection on genes associated with nutrient metabolism [10]. Clearly, changes at both sequence and expression level of genes related to nutrient metabolism play a key role in high-altitude adaptation of toads.

Changes associated with cardiac function may also play an important role. Heart displayed a large number of plastic DEGs (Table 1), which is not particularly surprising considering heart can adjust quickly to match metabolic demand [31]. It is, however, very interesting that the majority of these DEGs are down-regulated in high altitude individuals. In particular, genes related to cellular respiration and mitochondrion are mostly down-regulated (Fig. 2a). This may represent yet another significant difference from homeotherms. Previous work on skeletal muscle of homeotherms revealed an up-regulating trend for energy metabolism related genes [12, 21]. The heart of homeotherms often displayed an increased capacity at high-altitudes, which may facilitate the oxygen transport under hypoxia to sustain their normal metabolism [3]. Our results suggest that poikilotherms may have adopted different strategies to survive in high-altitude environments. We also detected some fixed DEGs in heart, which were mostly associated with muscle contraction, and the majority of these DEGs were also down-regulated (Additional file 2). The impact of these broad spectrum gene down-regulation on phenotype is unclear; however, our preliminary observation revealed that native high-altitude (Zoige) toads have much smaller hearts (heart length/ snout-vent length = 0.1557, S.D. = 0.0063, *n* = 6) than low-altitude (Chengdu) toads (0.1801, S.D. = 0.0137, *n* = 6).

Differing from liver and heart, brain did not show a broad spectrum of gene down-regulation. In fact, there are slightly more up-regulated genes than down-regulated ones in high altitude individuals in the two categories of response to stimulus (Fig. 3a). At high altitudes, environment is much more variable and much more extreme than at low altitudes. The observed regulatory changes of the brain are likely related to these amplified stimuli generated by the highly variable environment.

There are several limitations to our study. First, we did not conduct biochemical or physiological functional tests, and how these observed expression differences may translate into phenotypic variations remains unclear. However, previous phenotypic evidence is consistent with our results. Poikilothermic vertebrates including amphibians are known to suppress their metabolism to survive under hypoxia and hypothermia [22]. Amphibians at high-altitudes typically exhibited a decrease in growth rate compared to their low-altitude relatives [24, 32–34], which often indicates low metabolic rate and nutrition supply [32, 35]. Other poikilothermic vertebrates, e.g. toad-headed lizards (*Phrynocephalus erythrurus*), also showed a declined respiratory metabolism at high altitudes than at low altitudes [36]. The detected widespread down-regulation of genes associated with nutrient metabolism and cardiac function are likely associated with the above changes of phenotypes. Second, we had small sample sizes and our results should be considered preliminary. Only two egg masses were used in our comparison. Some of the observed differences could be difference between families rather than populations.

## Conclusion

Asiatic toads have clearly demonstrated both fixed and plastic changes in gene expression during their adaptation to high altitude environments, but the direction of expression regulation in the major modules is likely different from (and sometimes opposite to) that of homeotherms. A clear next step is to link the observed genetic level differences to phenotypic variation and fitness differences. Also, with this background knowledge, we now can move on to examine local adaptation and parallel changes among different populations. Poikilothermic vertebrates are among the most hypoxia-tolerant animals known, and many molecular mechanisms remain elusive. We hope that our results will provide useful directions for future studies.

## Additional files

Additional file 1: Summary information of digital gene expression profiles. (DOCX 84 kb)

Additional file 2: List of differentially expressed genes (DEGs) between toads from high- and low-altitude sites in three tissues (brain, heart, and liver) and the Gene Ontology (GO) categories over-represented by these genes. DEGs include both fixed and plastic, which were determined by a reciprocal transplant experiment. (XLSX 152 kb)

## Abbreviations

ACTC1: Actin, alpha, cardiac muscle 1
DEG: Differentially expressed gene
FDR: False discovery rate
GO: Gene Ontology
KEGG: The Kyoto Encyclopedia of Genes and Genomes
MED24: Mediator complex subunit 24
MYBPC2: Myosin-binding protein C, fast-type
OXPHOS: Oxidative phosphorylation
TNNC1: Troponin C type 1
