# Supplementary material for: Gene expression variations in high-altitude adaptation: a case study of the Asiatic toad (Bufo gargarizans)
Source: BMC Genet. 2017 Jul 3;18:62. doi: 10.1186/s12863-017-0529-z (PMC5496230; doi:10.1186/s12863-017-0529-z)
Supplement: Supplementary file 1 — Summary information of digital gene expression profiles. (DOCX 84 kb) [file 12863_2017_529_MOESM1_ESM.docx]

**Additional file 1.** Summary information of digital gene expression profiles.

| Sample | Tissue | Origin  site | Foster  site | Raw  Reads | Clean  reads | Clean bases | Error  (%) | Q20  (%) | GC  (%) |
| --- | --- | --- | --- | --- | --- | --- | --- | --- | --- |
| A-1 | liver | low | low | 15,937,614 | 15,895,808 | 0.79G | 0.01 | 98.75 | 45.2 |
| A-2 | heart | low | low | 10,988,096 | 10,968,168 | 0.55G | 0.01 | 98.71 | 46.34 |
| A-3 | brain | low | low | 11,949,072 | 11,922,601 | 0.6G | 0.01 | 98.63 | 45.93 |
| B-1 | liver | high | low | 12,907,777 | 12,882,759 | 0.64G | 0.01 | 98.74 | 45.03 |
| B-2 | heart | high | low | 9,945,589 | 9,929,748 | 0.5G | 0.01 | 98.67 | 46.29 |
| B-3 | brain | high | low | 11,320,913 | 11,300,146 | 0.57G | 0.01 | 98.59 | 45.74 |
| C-1 | liver | low | high | 11,090,695 | 11,036,653 | 0.55G | 0.01 | 98.73 | 45.7 |
| C-2 | heart | low | high | 11,366,371 | 11,345,841 | 0.57G | 0.01 | 98.46 | 46.93 |
| C-3 | brain | low | high | 11,589,195 | 11,565,961 | 0.58G | 0.01 | 98.64 | 45.97 |
| D-1 | liver | high | high | 13,193,277 | 13,159,345 | 0.66G | 0.01 | 98.49 | 46.1 |
| D-2 | heart | high | high | 15,418,938 | 15,383,048 | 0.77G | 0.01 | 98.41 | 47.29 |
| D-3 | brain | high | high | 11,678,000 | 11,648,776 | 0.58G | 0.01 | 98.41 | 46.06 |
